# Efficacy and Safety of the TCM Qi-Supplementing Therapy in Patients with Myasthenia Gravis: A Systematic Review and Meta-Analysis

**DOI:** 10.1155/2017/6512572

**Published:** 2017-12-03

**Authors:** Xi-qian Yang, Ling Liu, Wen-yu Yang, Huan-huan Dong, Yi-ran Yang, Yun Li

**Affiliations:** ^1^The TCM Clinical Institute, Hubei University of Chinese Medicine, Hubei 430065, China; ^2^The First Clinical Institute, Hubei University of Chinese Medicine, Hubei 430065, China; ^3^Encephalopathy Department, Hubei Provincial Hospital of TCM, Hubei 430061, China; ^4^The Second Clinical Medical College, Wenzhou Medical University, Zhejiang 325000, China

## Abstract

**Background:**

The Traditional Chinese Medicine (TCM) Qi-supplementing therapy has been used widely for treating myasthenia gravis (MG) in China. The purpose of this meta-analysis was to evaluate the efficacy and safety of Qi-supplementing therapy as an adjunctive therapy in MG patients.

**Methods:**

Seven electronic databases were searched through June 2016. Randomized controlled trials (RCTs) evaluating the add-on effect of Qi-supplementing therapy in MG patients were included. The outcome measures were the total effective rate, relapse rate, and adverse events.

**Results:**

Twenty-three RCTs involving 1,691 MG patients were included. The included studies were of low-to-moderate quality. Meta-analysis showed that Qi-supplementing therapy combined with Western medicine (WM) significantly improved the total response rate and reduced the relapse risk during 6–24 months of follow-up. Subgroup analysis showed that Qi-supplementing therapy only affected the total response rate within the first 6 months of treatment. Moreover, the rate of adverse events was lower with the addition of Qi-supplementing therapy to WM than with WM only.

**Conclusions:**

Short-term Qi-supplementing therapy combined with WM appears to be superior to WM for improving the total response rate and reducing the relapse rate. However, more high-quality RCTs are warranted owing to methodological flaws of previous trials.

## 1. Introduction

Myasthenia gravis (MG) is a chronic autoimmune disorder of the neuromuscular junction of the skeletal muscle that is characterized by fluctuating pronounced ocular, limb muscle, or bulbar weakness [1]. MG is a global public health problem due to its increasing incidence [2]. The reported incidence of MG has markedly varied across epidemiological studies, and the pooled annual incidence of MG was calculated to be 5.3 per million persons based on 55 population-based epidemiological studies [3]. Notably, MG diagnosis is associated with increased risk of mortality [4, 5].

Current therapeutic strategies mainly include acetylcholinesterase inhibitors, immunosuppressive agents, and thymectomy [6]. Pyridostigmine is recommended as a first-line anticholinesterase inhibitor for acute exacerbations [7]. Long-term corticosteroids and azathioprine still remain the first choice for patients with severe or refractory disease [8]. Conventional treatment can effectively control symptoms in most patients. Unfortunately, a small proportion of cases still have poor disease control or require high-dose immunosuppressive agents [9, 10]. In addition, adverse effects induced by long-term use of steroids or other immunosuppressive agents remain an important concern [11]. Therefore, alternative therapies with better efficacy and fewer side effects are urgently needed.

MG is generally diagnosed as “flaccidity syndrome” in Traditional Chinese Medicine (TCM). According to the TCM theory, the syndrome is thought to be caused by Qi-deficiency [35]. Treatment with Qi-supplementing Formula in combination with the conventional Western medicine (WM) has achieved promising clinical effects in terms of better clinical efficacy and fewer adverse effects [36, 37]. However, the strength of this conclusion is limited by the small sample sizes of most trials. In addition, no previous systematic review or meta-analysis has evaluated the add-on effect of Qi-supplementing Formula for treating MG.

In the current study, we conducted a systematic review and meta-analysis to evaluate the efficacy and safety of Qi-supplementing Formula as an adjunctive therapy in MG patients based on available randomized controlled trials (RCTs).

## 2. Methods

### 2.1. Study Selection

This study was conducted according to the Preferred Reporting Items for Systematic Reviews and Meta-Analyses (PRISMA) guidelines [38]. Trials were eligible if they satisfied the following criteria: (1) RCTs evaluating the efficacy and safety of Qi-supplementing Formula treatment for MG; (2) patients with MG diagnosed according to the accepted diagnosis criterion, irrespective of gender, age, or ethnicity; (3) treatment group receiving any Qi-supplementing Formula plus WM treatment (cholinesterase inhibitors, immunosuppressive agents, or thymectomy) and having been compared with a control group treated with WM alone; and (4) outcome measures including total effective rate, relapse rate, and adverse events. The efficacy of the intervention was reported across four categories: clinically cured, markedly effective (the effective rate was defined as the number of patients who achieved a clinical cure), markedly effective, and effective divided by the total number of patients. Disease recurrence was defined as return of the disease during follow-up after drug withdrawal. Trials were excluded according to the following criteria: (1) a nonrandomized trial or self-control study; (2) sample size less than 30 cases; (3) Qi-supplementing Formula given in combination with acupuncture, acupoint injection, massage, or rehabilitation therapy as intervention; (4) patients with a MG crisis; and (5) trial with missing data or duplicated publication.

### 2.2. Search Strategy

Two reviewers independently searched the following databases for RCTs from their inception to June 2016: China National Knowledge Infrastructure (CNKI), China Biological Medicine (CBM), Chinese Scientific Journals Database (VIP), Wanfang database, PubMed, EMBASE, and Cochrane Library. The following search terms were used in combination: (Buqi OR Yiqi OR Qi-supplementing) AND (myasthenia gravis OR flaccidity OR paralysis OR Weizheng) AND (Traditional Chinese Medicine OR TCM) AND (randomized OR random OR randomized controlled trials OR RCTs). The reference lists of the retrieved articles and relevant reviews were manually searched to identify any additional eligible trials. We also searched conference proceedings and dissertations to identify unpublished trials. Only trials published in Chinese and English were considered.

### 2.3. Data Extraction and Quality Assessment

Two reviewers independently extracted the data from the selected trials into a standard data extract form. The extracted data included first authors' name, publication year, sample size, age of participants, interventions, durations of treatment and follow-up, and outcome measures. We evaluated the methodological quality of the included trials in accordance with the Cochrane risk of bias tool [39]. The judgment of risk of bias includes random sequence generation, allocation concealment, blinding of participants and personnel, blinding of outcome assessments, incomplete outcome data, selective reporting, and other sources of bias. Any disagreements were resolved by discussion with a third reviewer.

### 2.4. Statistical Analysis

The pooled summary was expressed as risk ratio (RR) with 95% confidence interval (CI) for discontinuous variables. Heterogeneity across trials was tested using the *I*^2^ statistic and Cochrane *Q* statistic. If *I*^2^ > 50% and *P* > 0.10 for the Cochrane *Q* statistic, we selected a random effect model; otherwise, a fixed effect model was applied. Subgroup analysis was performed based on the different treatment durations. To assess potential publication bias, a funnel plot was carried out and generated. All statistical analyses were conducted using Review Manage software 5.0 (Cochrane Collaboration, Oxford, UK) and STATA statistical software 12.0 (STATA Corp LP, College Station, TX, USA).

## 3. Results

### 3.1. Search Results and Trial Characteristics

**Figure 1:**
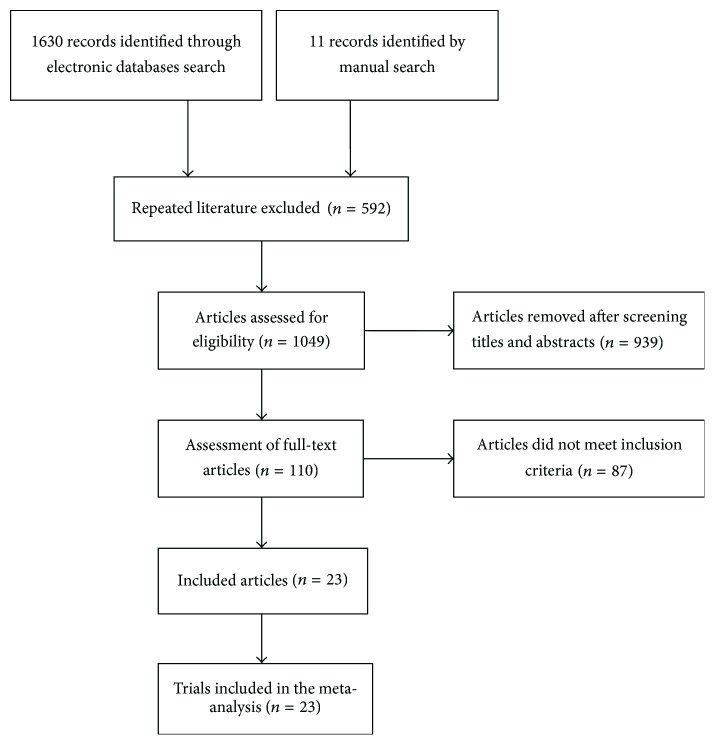
Flow chart of trial selection process.

A total of 1,641 records were retrieved from the above-mentioned databases and through the manual literature search. Of these, 592 articles were excluded upon exclusion of duplicated publications. After reading the titles and abstracts, another 929 articles were removed. Thus, 110 full-text articles were assessed for eligibility. After a detailed assessment of the full-text papers, an additional 87 articles were excluded mainly because they did not satisfy our predefined inclusion criteria. Finally, 23 RCTs [12–34] were included in the meta-analysis (Figure 1).

Table 1 presents the baseline characteristics of patients in the trials included in the meta-analysis. All the included trials were carried out in China and published from 1999 to 2015. Apart from one trial [34] published in English, all others were published in Chinese journals. The sample sizes ranged from 36 to 212. A total of 1,691 patients were included in the 23 trials. The Qi-supplementing Formula group consisted of 882 patients, while the WM treatment group consisted of 809 patients. Of the WM treatments, 4 trials [14, 24, 30, 32] used only cholinesterase inhibitors, 7 trials [13, 15, 16, 22, 26, 27, 29, 33] applied immunosuppressants alone, 10 trials [12, 17–21, 23, 25, 28, 31] used cholinesterase inhibitors in combination with immunosuppressants simultaneously, and 1 trial [34] used two types of immunosuppressants. The course of treatment ranged from 1 to 24 months. For all cases, the Qi-supplementing Formula included Radix Astragali. The modified Bu Zhong Y Qi decoction/pill was used in 12 trials [15, 17–20, 24, 26, 27, 30, 33, 34].

### 3.2. Methodological Quality of the Included Trials

**Figure 2:**
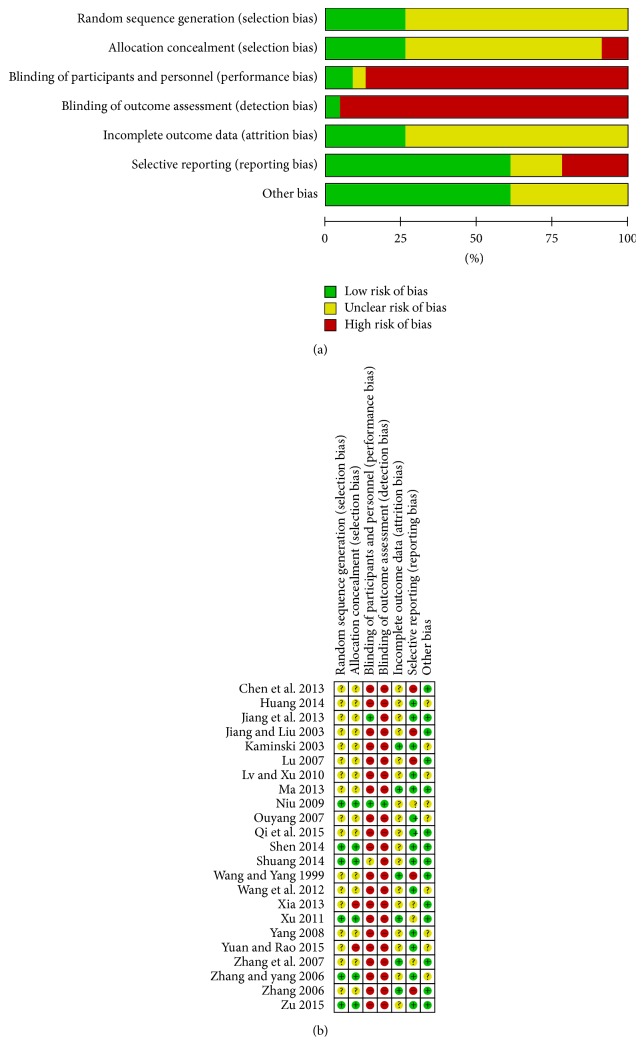
Graph of bias risk (a) and summary of bias risk (b).

Figure 2 summarizes the methodological quality of the 23 RCTs. Six trials [16, 21, 23, 29, 31, 33] were randomized using random number tables to generate a sequence (appropriate), two trials [28, 32] used a temporal sequence for randomization (inappropriate), and the remaining trials only mentioned randomization without detailed methods. Three trials [21, 25, 31] were randomized controlled placebo designs. One trial [21] reported the blinding of outcome assessments, and one trial [34] reported the patients' reasons for withdrawal or loss to follow-up.

### 3.3. Total Clinical Effective Rate

**Figure 3:**
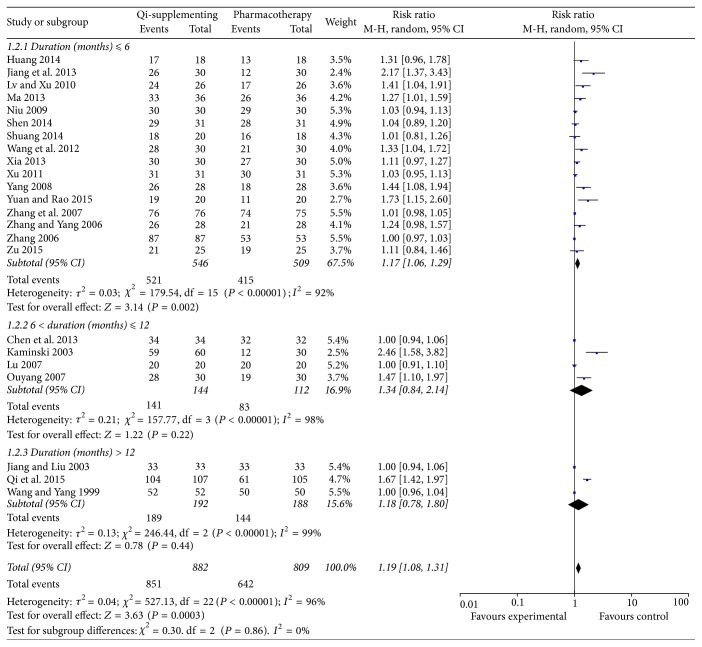
Forest plots showing risk ratio with 95% confidence interval for the total effective rate comparing treatments with or without Qi-supplementing Formula therapy in a random effect model.

All the trials reported the total effective rate as an outcome. As shown in Figure 3, significant heterogeneity (*I*^2^ = 96%, *P* < 0.001) between trials was observed, and, thus, we selected a random effect model. Overall, Qi-supplementing Formula in combination with WM treatment could improve the total effective rate by 19% (RR 1.19; 95% CI 1.08–1.31; *P* = 0.0003) compared with WM treatment alone. A subgroup analysis was implemented according to the duration of treatment. The results showed that short-term (less than 6 months) treatment with Qi-supplementing Formula significantly improved the total effective rate (RR 1.17; 95% CI 1.06–1.29; *P* = 0.002). However, the total effective rates from 6 to 12 months (RR 1.34; 95% CI 0.84–2.14; *P* = 0.22) and beyond 12 months (RR 1.18; 95% CI 0.78–1.80; *P* = 0.44) of treatment with Qi-supplementing Formula in combination with WM were not improved over those achieved with WM treatment alone.

### 3.4. Relapse Rate

**Figure 4:**
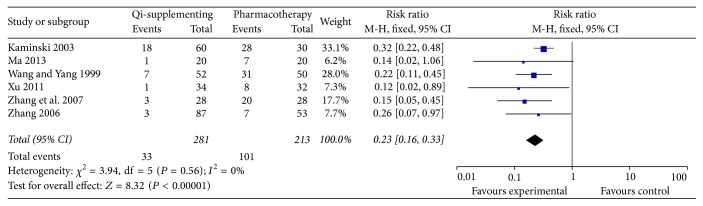
Forest plots showing risk ratio with 95% confidence interval for the relapse rate comparing treatments with or without Qi-supplementing Formula therapy in a fixed effect model.

Nine trials [12, 14, 15, 19, 20, 22, 23, 26, 33] included relapse events as an outcome. However, three trials [20, 22, 33] did not provide the detailed numbers of relapse events. Therefore, only six trials [12, 14, 15, 19, 23, 26] were included in the pooled analysis. Recurrence events were recorded in 33/180 patients in the Qi-supplementing Formula plus WM treatment group compared with 101/213 patients in the WM treatment only group. As shown in Figure 4, there was no significant heterogeneity across the trials (*I*^2^ = 0%, *P* = 0.56). Meta-analysis showed that Qi-supplementing Formula in combination with WM treatment was associated with a significantly lower risk of relapse events (RR 0.23; 95% CI 0.16–0.33; *P* < 0.01) in a fixed effect model.

### 3.5. Adverse Events

Fourteen trials [12, 13, 15, 17, 19, 23, 25–29, 31, 33, 34] reported adverse events. Of these, 1 trial [34] reported 8 cases of withdrawal due to inability to tolerate myelosuppression and other adverse reactions; 2 trials [12, 19] mentioned adverse events but did not give detailed descriptions; and 2 trials [17] described the symptomatic adverse reaction to the TCM. The remaining 10 trials provided the details for adverse events. Fifty-six patients developed adverse events in the Qi-supplementing Formula plus WM treatment group compared with 155 patients in the WM treatment only group. The most common adverse events included gastrointestinal reactions (63 cases), glucocorticoid-induced obesity (33 cases), rash (13 cases), abnormal liver function (9 cases), leukopenia (9 cases), thrombocytopenia (11 cases), femoral head necrosis (5 cases), elevated serum creatinine (7 cases), hyperglycemia (9 cases), infection (7 cases), osteoporosis (3 cases), and hypertension (1 case).

### 3.6. Publication Bias

**Figure 5:**
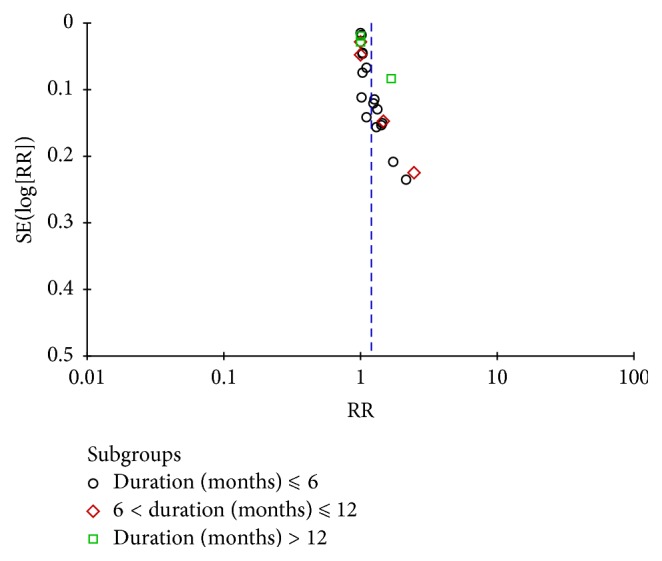
Funnel plots based on the total effective rate.

A publication bias test was performed for the total effective rate, which included more than 10 trials. The shape of the funnel plot showed slight asymmetries, indicating a possible publication bias (Figure 5).

## 4. Discussion

To the best of our knowledge, this is the first systematic review and meta-analysis to evaluate the efficacy and safety of Qi-supplementing Formula as an adjunctive therapy for the treatment of MG. The main findings of the present meta-analysis were as follows: (1) Qi-supplementing Formula combined with WM could improve by the total effective rate of symptom improvement by 19% compared with WM treatment alone; (2) compared with WM treatment, Qi-supplementing Formula therapy was associated with a 77% lower risk of relapse; and (3) adjunctive treatment with Qi-supplementing Formula appeared to be well tolerated and safe in patients with MG. This meta-analysis suggests that Qi-supplementing Formula therapy may have beneficial effects in the management of MG patients.

Subgroup analysis on the course of treatment revealed that short-term (within 6 months) treatment with Qi-supplementing Formula significantly improved the total effective rate. However, the treatment effects in the middle term (6–12 months) and long term (>12 months) did not differ significantly between WM combined with Qi-supplementing Formula therapy and WM alone. However, these findings may be explained by a lack of statistical power due to the small number of patients included in the analysis.

Our study is different from previous published meta-analyses. A previously well-designed meta-analysis [40] suggested that TCM as a whole combined with WM appeared to be an effective intervention in the treatment of MG compared with WM alone. However, this meta-analysis did not specifically focus on Qi-supplementing Formula. Another meta-analysis [41] indicated that TCM combined with WM could improve the total effective rate and reduce acetylcholine receptor antibodies levels in MG patients. With subgroup analyses by treatment duration and relapse of disease as outcome measures, our meta-analysis provided stronger evidence for the use of Qi-supplementing Formula in the management of MG.

Apart from the clinical efficacy, the adverse events after treatment with Qi-supplementing Formula combined with WM were an important concern. Gastrointestinal reactions and glucocorticoid-induced obesity were frequently recorded adverse events in our meta-analysis. Glucocorticoid-induced obesity, infection, and femoral head necrosis could not be attributed to the Qi-supplementing Formula but were known side effects of corticosteroids. The most frequently used herb in the included RCTs was Radix Astragali. Radix Astragali is a widely used herb for Tonifying-Qi in TCM. Gastrointestinal reactions may be partly explained by a large dose of Radix Astragali. However, Qi-supplementing Formula appeared to reduce the occurrence of adverse events in our study. The total number of adverse events recorded was lower for treatment with Qi-supplementing Formula plus WM than with WM alone. Therefore, Qi-supplementing Formula seemed to be well tolerated and safe in patients with MG. However, it should be noted that inadequate reporting on adverse events in the included trials was a major concern.

The current meta-analysis has several limitations. First, the methodological quality of the included trials was generally substandard. Although all included trials stated the use of randomization, most of the trials lacked a description of an adequate random allocation method, allocation concealment or blinding, and withdrawal or loss of follow-up. Second, substantial heterogeneity was observed in the pooled total effective rate. The reason for the heterogeneity may be correlated with the use of different types of WM, diverse herbs used in the Qi-supplementing Formula, and a wide range of interventions. Third, all included RCTs were carried out in China, and only one trial was published in English and indexed in the PubMed. Thus, potential publication bias may exist. Finally, most of included trials did not consider syndrome differentiation in the enrollment of MG patients. Qi-supplementing Formula in combination with WM treatment for MG should be based on the dual diagnosis. Selection of inappropriate patients may have decreased the actual efficacy of Qi-supplementing Formula.

Several issues should be addressed to improve the methodological quality in future trials. First, RCTs evaluating the efficacy and safety of Qi-supplementing Formula should follow the international standards such as PRISMA guidelines, which can be helpful for improving the quality of trials. Second, treatment based on syndrome differentiation is the core of TCM. Using TCM syndrome differentiation is the key to enhancing the efficacy of treatment. Finally, a sufficient follow-up duration is warranted to evaluate the long-term efficacy of Qi-supplementing Formula due to the relapse nature of MG.

## 5. Conclusions

The current best evidence demonstrates that short-term adjuvant treatment with Qi-supplementing Formula in addition to conventional WM treatment appears to have favorable effects on improving the total effective rate and reducing the risk of relapse. However, our conclusions should be interpreted with caution due to the poor methodological quality of the included trials and the high heterogeneity. More high-quality RCTs with large sample sizes are needed to verify the current findings.

## Figures and Tables

**Table 1:** Baseline characteristics of the included trials.

Author/year	Region	Number of participants	Age range/mean (years)	Intervention	Treatment duration	Outcome measures	Follow-up duration	TCM pattern differentiation
Wang and Yang 1999 [12]	China	Q: 52 C: 50	7–45	Q: Qi-supplementing Formula + controls; C: cholinesterase inhibitors + glucocorticoid	24 months	① + ②	24 months	No
Jiang and Liu 2003 [13]	China	Q: 33 C: 33	Q: 36.5 C: 35	Q: Qi-supplementing Formula + controls; C: immunosuppressant	12–24 months	① + ③	Not mentioned	No
Kaminski 2003 [14]	China	Q: 60 C: 30	20–61	Q: Qi-supplementing Formula + controls; C: pyridostigmine	9 months	① + ②	6 months	No
Zhang 2006 [15]	China	Q: 87 C: 53	3–16 (9.6)	Q: Qi-supplementing Formula + controls; C: prednisone	4 months	① + ② + ③	12 months	No
Zhang and Yang 2006 [16]	China	Q: 28 C: 28	Q: 31.1 C: 32.2	Q: Qi-supplementing Formula + controls; C: prednisone	6 months	①	Not mentioned	No
Lu 2007 [17]	China	Q: 20 C: 20	Q: 10–53 C: 15–49	Q: Qi-supplementing Formula + controls; C: glucocorticoid + pyridostigmine	12 months	①	Not mentioned	Yes
Ouyang 2007 [18]	China	Q: 30 C: 30	Q: 28.2 C: 27.5	Q: Qi-supplementing Formula + controls; C: prednisone + pyridostigmine	10–12 months	①	Not mentioned	No
Zhang et al. 2007 [19]	China	Q: 76 C: 75	1.5–12 (6.8)	Q: Qi-supplementing Formula + controls; C: prednisone + pyridostigmine	4.5–6 months	① + ②	12 months	No
Yang 2008 [20]	China	Q: 28 C: 28	Q: 9–69 C: 8–70	Q: Qi-supplementing Formula + controls; C: prednisone + pyridostigmine	3 months	①	3 months	Yes
Niu 2009 [21]	China	Q: 30 C: 30	Q: 43.2 ± 18.6 C: 43.2 ± 17.14	Q: Qi-supplementing Formula + controls; C: prednisone + pyridostigmine + placebo	3 months	①	Not mentioned	Yes
Lv and Xu 2010 [22]	China	Q: 26 C: 26	Q: 12–60 C: 10–58	Q: Qi-supplementing Formula + controls; C: prednisone	3–6 months	①	3 months	Yes
Xu 2011 [23]	China	Q: 31 C: 31	39–73 (62.5)	Q: Qi-supplementing Formula + controls; C: prednisone + pyridostigmine	≥4.5 months	① + ② + ③	12 months	No
Wang et al. 2012 [24]	China	Q: 30 C: 30	20–60	Q: Qi-supplementing Formula + controls; C: pyridostigmine	6 months	①	Not mentioned	Yes
Jiang et al. 2013 [25]	China	Q: 30 C: 30	Q: 17–75 (48.7) C: 22–75 (49.0)	Q: Qi-supplementing Formula + controls; C: prednisone + pyridostigmine + placebo	6 months	① + ③	Not mentioned	Yes
Ma 2013 [26]	China	Q: 36 C: 36	Q: 2–12 C: 3–12	Q: Qi-supplementing Formula + controls; C: prednisone	3 months	① + ② + ③	Not mentioned	No
Chen et al. 2013 [27]	China	Q: 34 C: 32	Q: 20.8 C: 21.2	Q: Qi-supplementing Formula + controls; C: prednisone	12 months	① + ③	Not mentioned	Yes
Xia 2013 [28]	China	Q: 30 C: 30	14–72 (34.6)	Q: Qi-supplementing Formula + controls; C: prednisone + pyridostigmine	2 months	① + ③	Not mentioned	Yes
Shen 2014 [29]	China	Q: 31 C: 31	32–60	Q: Qi-supplementing Formula + controls; C: glucocorticoid	1 month	① + ③	Not mentioned	No
Huang 2014 [30]	China	Q: 18 C: 18	18–60	Q: Qi-supplementing Formula + controls; C: pyridostigmine	6 months	①	Not mentioned	No
Shuang 2014 [31]	China	Q: 20 C: 18	Q: 22–60 C: 20–59	Q: Qi-supplementing Formula + controls; C: prednisone + pyridostigmine + placebo	2.5 months	① + ③	Not mentioned	Yes
Yuan and Rao 2015 [32]	China	Q: 20 C: 20	Not reported	Q: Qi-supplementing Formula + controls; C: pyridostigmine	2.25 months	①	Not mentioned	Yes
Zu 2015 [33]	China	Q: 25 C: 25	13–66 (32.4)	Q: Qi-supplementing Formula + controls; C: prednisone	2 months	① + ③	1 month	Yes
Qi et al. 2015 [34]	China	Q: 107 C: 105	16–73	Q: Qi-supplementing Formula + controls; C: methylprednisolone + azathioprine	18 months	① + ②	Not mentioned	No
